# Radiopaque Fully Degradable Nanocomposites for Coronary Stents

**DOI:** 10.1038/s41598-018-35663-2

**Published:** 2018-11-27

**Authors:** Hui Ying Ang, Daniel Toong, Wei Shoon Chow, Welly Seisilya, Wei Wu, Philip Wong, Subbu S. Venkatraman, Nicolas Foin, Yingying Huang

**Affiliations:** 1 National Heart Centre Singapore, 5 Hospital Drive, 169609 Singapore, Singapore; 2 School of Materials Science and Engineering, Nanyang Technological University, Nanyang Avenue, 639798 Singapore, Singapore; 3 Department of Mechanical Engineering, University of Texas at San Antonio, 1 UTSA Circle, San Antonio, TX 78249 USA; 4 Duke-NUS Medical School, 8 College Road, 169857 Singapore, Singapore

## Abstract

Bioresorbable scaffolds (BRS) were introduced to overcome limitations of current metallic drug-eluting stents and poly-L-lactide (PLLA) has been used in the fabrication of BRS due to its biodegradability and biocompatibility. However, such polymers have weaker mechanical properties as compared to metals, limiting their use in BRS. We hypothesized that nanofillers can be used to enhance the mechanical properties considerably in PLLA. To this end, polymer-matrix composites consisting of PLLA reinforced with 5–20 wt% barium sulfate (BaSO_4_) nanofillers as a potential BRS material was evaluated. Stearic-acid (SA) modified BaSO_4_ nanofillers were used to examine the effect of functionalization. Rigid nanofillers improved the tensile modulus and strength of PLLA (60% and 110% respectively), while the use of SA-BaSO_4_ caused a significant increase (~110%) in the elongation at break. Enhancement in mechanical properties is attributed to functionalization which decreased the agglomeration of the nanofillers and improved dispersion. The nanocomposites were also radiopaque. Finite element analysis (FEA) showed that scaffold fabricated from the novel nanocomposite material has improved scaffolding ability, specifically that the strut thickness could be decreased compared to the conventional PLLA scaffold. In conclusion, BaSO_4_/PLLA-based nanocomposites could potentially be used as materials for BRS with improved mechanical and radiopaque properties.

## Introduction

Current metallic drug-eluting stents (DES) have thin struts with biocompatible polymer coating, making them the first choice device for the treatment of coronary artery disease. However, the caging of the vessel permanently with a metallic implant runs the risk of impairing endothelial function and decreasing positive lumen remodeling. Clinical observations from several large-scale DES registries have revealed the occurrence of late adverse events that questions the safety of DES. Hence, bioresorbable scaffolds/stents (BRS) present an attractive alternative to DES by providing the temporal support and be resorbed in due time, allowing the vessel to return to a more natural and healed state^1,2^.

Conceptually, the BRS is supposed to retain sufficient radial strength after implantation to prevent vessel recoil and to release the antiproliferative drug. After the healing period, the BRS is no longer required and should degrade to be resorbed completely, leaving the vessel with a healthy endothelium and normal vasomotion. BRS fabricated from bioresorbable polymeric materials are, in theory, more flexible and conformable and would influence the shear stress pattern to a lesser degree compared to a metallic DES^3,4^. The absence of any residual foreign material and restoration of endothelial coverage would also reduce the need for long-term dual antiplatelet treatment thereby decreasing the risk of bleeding. The resorption of the BRS would allow future intervention at the same site and facilitate the access to side branches that were jailed by the original stent^5,6^. Current BRS are either polymer-based (e.g. Poly-L-lactide, Polycarbonate) or metallic-based (e.g. Magnesium alloy).

Poly-L-lactide (PLLA) is a biodegradable, biocompatible and biologically inert synthetic polymer used widely in biomedical application such as tissue engineering scaffolds. PLLA-based BRS include ABSORB Bioresorbable Vascular Scaffold (BVS) (Abbott Vascular), DESolve (Elixir Medical), FORTITUDE/APTITUDE/MAGNITUTDE (Amaranth), ArterioSorb (Arterius), MIRAGE (Manli Cardiology), ART bioresorbable scaffolds (Arterial Remodeling Technologies). Biotronik’s Magmaris is the only resorbable metallic BRS with a CE mark^1^.

PLLA has been widely studied as a biomaterial for several biomedical engineering applications due to its biodegradability and biocompatibility^7–9^. Particularly, PLLA has been investigated widely as a choice of material in BRS fabrication^10^. Unprocessed PLLA will typically exhibit approximately 100-fold lower tensile modulus than cobalt or stainless steel which are the conventional materials used in DES. As a consequence of the lower modulus (both tensile and compressive), BRS fabricated from these bioresorbable materials may require up to 240% thicker struts in order to match the radial strength of the current metallic DES, thereby affecting the device’s deliverability^11^.

Polymeric devices also have a limit of expansion and can fracture due to over-dilation. It is important to improve the expandability of the BRS while maintaining radial strength^12^. With lesser strength, BRS requires extensive vessel preparation and may achieve lesser acute gain (defined as the difference between pre-procedural minimal luminal diameter, MLD, and immediate post-procedural MLD) than a metallic stent. Current PLLA devices also lack radiopacity, making the visualization and assessment of scaffold expansion difficult^1,13^.

Nanocomposite polymeric materials present a novel class of materials with important properties in several engineering and biomedical applications. A polymeric nanocomposite comprises nanofillers dispersed within a polymer matrix. The concept of a nanocomposite material capitalizes on the inherent properties of the base polymer while enhancing the functionality of the composite device by the addition of nanofillers^14,15^. The nanofillers are able to render additional features to the polymer that are usually not available in polymeric materials such as optical, electrical and mechanical properties^16–18^. In the field of biomaterials, the reinforcement effect of rigid nanofillers on polymers’ mechanical properties is of particular interest. Nanofillers possess a large surface to volume ratio that increases the number of particle-matrix interactions when dispersed in a polymer and can improve the overall material properties^19–22^. The presence of the filler can reinforce the mechanical strength of the polymer by: (i) substituting the softer polymer matrix by a stiffer filler, (ii) immobilizing the polymer molecules on filler particle surfaces as a result of filler–polymer interaction, and (iii) stress transfer from the matrix to the filler^23^. The nanofillers can help to absorb energy from the applied stress and disperse it about a larger volume of the nanocomposite material, thereby improving the composite’s properties^24,25^.

The reinforcement effect of the nanofillers on the nanocomposite properties depends not just on the nature of fillers and polymer but also on the filler size, filler-polymer interfacial interaction and particle loading. It is well documented that the major challenge in fabricating nanocomposites is achieving a uniform dispersion and good integration of the nanofillers in the polymer matrix^26–28^. Most of the polymers are hydrophobic and incompatible with the hydrophilic nanofillers. The issue of nanofiller agglomeration and weak particle-polymer affinity often lead to detrimental effects on the mechanical strength^29–31^. The interactions between the polymer and filler at the interface significantly influence the composite’s mechanical properties. Interfacial debonding is commonly the first step of failure and a lack of adhesion between the two phases will lead to phase separation and early material failure^32^. In the same way, a strong interfacial bonding between the nanofillers and polymer leads to an effective transfer of load from the matrix to the nanofillers, enhancing the composite’s strength.

To achieve good interfacial adhesion between the fillers and polymer matrix, functionalization is often utilized to improve the particle-polymer affinity. Functionalization of the nanofillers can help to establish favorable interactions that prevent agglomeration of the fillers and improve the overall distribution of the fillers within the polymer matrix^33–35^. One method to functionalize the nanofillers is to covalently graft another suitable compound such as a surfactant to the surface of the fillers before addition to the polymer and this has yielded positive mechanical outcomes for bone cement applications^36,37^.

In this study, we hypothesize that the use of PLLA reinforced by the addition of inorganic nanofillers such as barium sulfate (BaSO_4_) can potentially overcome the drawbacks of current PLLA as a load-bearing biomaterial. BaSO_4_ has been used extensively as a radiocontrast agent in X-ray imaging and other diagnostic procedures. Several studies have demonstrated the reinforcement and radiopaque properties of BaSO_4_ in polymeric materials. Stearic acid was selected as a functionalizing agent due to its surfactant properties, enabling it to be conjugated to the inorganic fillers while forming targeted hydrophobic interaction with PLLA^38,39^. Hence in this study, we have formulated a nanocomposite material based on inorganic BaSO_4_ nanofillers and PLLA, and studied the effect of loading and functionalization on the mechanical properties of PLLA. Finite Element Analysis (FEA) was also carried out to evaluate the effect of the materials using a generic stent design, and the nanocomposite material was employed to improve the scaffolding while reducing the stent thickness potentially.

## Results

### Effect of filler loading on nanocomposite mechanical properties

**Figure 1:**
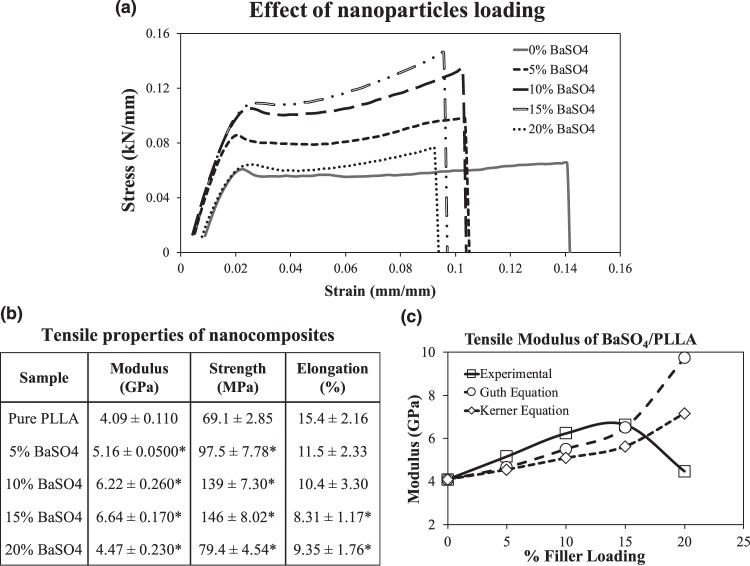
Tensile properties of nanocomposite BaSO_4_/PLLA fibers with different filler loadings (0–20%). (**a**) Representative stress-strain curve of the different nanocomposite samples. (**b**) Mean tensile modulus, strength and elongation at break of the nanocomposites are represented as mean value ± standard deviation. (n = 10) * indicates a statistically different value of the composite compared to the standard PLLA based on one-way ANOVA (p < 0.05). (**c**) The predicted tensile modulus of BaSO_4_/PLLA nanocomposite as a function of filler loading according to Guth’s equation and Kerner’s equation^40,42^.

The addition of non-functionalized BaSO_4_ nanofillers in PLLA has a significant effect on the polymer tensile properties (Fig. 1). As seen from Fig. 1a,b, as the amount of nanofillers increased from 0% to 15%, the modulus and ultimate strength of the material increased, thereby demonstrating the mechanical reinforcement effect of the rigid nanofillers. However, further increment in BaSO_4_ loading to 20% caused a significant decrease in the modulus and strength (p < 0.05). The optimal reinforcement results occurred at 15% BaSO_4_ loading with a 62% and 300% increase in tensile modulus and strength respectively. The presence of the BaSO_4_ nanoparticles caused a significant decrease in the material’s elongation at break across all filler loadings.

#### Tensile Modulus

It has been reported that the tensile modulus of polymers can be improved by adding nanofillers since rigid inorganic particles are much stiffer than polymer matrices. Two models that predict the reinforcing effect of fillers on the matrix have been used in this study to correlate the experimental observations and the reinforcement effect of the nanofillers. The Guth’s equation (Equation 1), which is a modified version of Einstein’s equation was employed^40^:

1 $${{\rm{E}}}_{{\rm{c}}}/{{\rm{E}}}_{{\rm{m}}}=1+2.5\,{{\rm{V}}}_{{\rm{p}}}+14.1{{{\rm{V}}}_{{\rm{p}}}}^{2}$$

where E_c_ and E_m_ are the tensile modulus of composite and matrix and V_p_ is the particle volume fraction (obtained based on the method described in Section 2.2). This equation is often used to estimate the effect of fillers on composite modulus as it incorporates: (1) a linear term reflects the stiffening effect of the fillers and (2) the second power term is the contribution of filler interaction, which is higher at increased filler loading^40,41^. According to Equation 1, increasing the particle volume fraction is expected to increase the modulus of the composite. Another model used to predict the effect of spherical nanofillers on composite material’s modulus is the Kerner’s equation (Equation 2)^42^:

2 $${{\rm{E}}}_{{\rm{c}}}/{{\rm{E}}}_{{\rm{m}}}=1+{{\rm{V}}}_{{\rm{p}}}{(1-{{\rm{V}}}_{{\rm{p}}})}^{-1}\,\ast \,15(1\,-\,{{\rm{v}}}_{{\rm{m}}}){(8-{{\rm{10v}}}_{{\rm{m}}})}^{-1}$$

where v_m_ is the matrix Poisson ratio and taken to be 0.475 according to Soares *et al*.^43^. Figure 1c shows the estimated effect of nanofillers loading on the composite’s modulus based on the two equations as compared to the experimental data.

From Fig. 1c, it is observed that BaSO_4_ nanofillers have a reinforcement effect on the PLLA matrix, as increasing the filler loading (up till 15% by weight) increased the modulus. This improvement in modulus is in agreement with the modelling equations used. The results demonstrated that the Guth’s model for predicting fillers effect on modulus is closer to the observed experimental data as compared to the Kerner’s equation. However, both models do not predict a maximum in the modulus vs. volume fraction curve, which was observed experimentally here. The lesser fit of the Kerner’s equation to the data can be attributed to its assumption that there is no interaction between particle–particle or matrix–particle, however, this will not be the case in the BaSO_4_/PLLA system as seen in the agglomeration of the nanofillers^44^.

#### Tensile Strength

Stress transfer between the fillers and the polymer affects the strength of the material. For nanocomposites with good interfacial adhesion, the applied stress can be transferred effectively from the polymer matrix to the fillers, which helps to improve the strength^41^. Nanofillers may also adversely affect the tensile strength of the composite material by acting as stress concentrators, due primarily to poor interfacial adhesion that results in poor or no stress transfer. There are many different equations and empirical models proposed to predict the effect of fillers on the composite tensile strength, but most models assume poor or no adhesion of nanofillers to matrix, which then predict a monotonic decrease in the composite strength with the addition of the fillers^45–47^. Interestingly, none of these models are applicable to this BaSO_4_/PLLA nanocomposite system as the experimental data led to an increase in ultimate tensile strength with the addition of the fillers, passing through a maximum at 15% filler loading (similar to tensile modulus).

**Figure 2:**
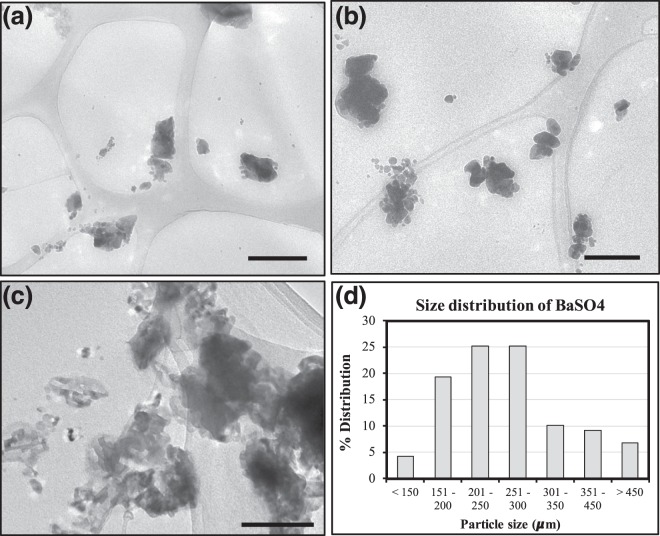
TEM images of BaSO4/PLLA nanocomposites with different filler loading at: (**a**) 5%, (**b**) 15% and (**c**) 20%. With increased filler loading, larger clusters of agglomerated BaSO_4_ nanofillers are observed. Scale bar = 200 nm for all images. (**d**) Size distribution of the agglomerated BaSO_4_ nanofillers based on n = 120 clusters/particles. The average particle size was 270 ± 105 µm in the 20% BaSO_4_/PLLA system.

The increase in tensile strength of the composites upon the addition of the rigid fillers affirmed some form of adhesion between the nanofillers and the matrix. However, increasing the filler loading also led to an increased agglomeration of the fillers in the matrix, which could explain the decreased mechanical properties at 20% BaSO_4_ loading. Nanosized fillers such as BaSO_4_ have high surface energies, causing them to aggregate in order to lower the surface energies^48^. Such agglomeration is observed in the TEM images of the nanocomposite fibers with different BaSO_4_ loading (Fig. 2). At the highest filler loading (20%), agglomeration is more prevalent as seen in Fig. 2c, leading to uneven distribution of fillers, which can result in phase separation, decreasing the mechanical properties. The size distribution of BaSO4 nanoparticles/clusters in the 20% BaSO_4_/PLLA system is shown in Fig. 2d, more than 50% of the agglomerated the fillers were in the 200–300 µm size range. This observation has been reported in several other nanofillers/polymer composite studies^49–52^.

#### Elongation at Break

The elongation at break of the nanocomposite material was significantly reduced when BaSO_4_ nanofillers were added to PLLA across all filler loading, indicating a decrease in material ductility (Fig. 1). This significant decrease in elongation at break of the nanocomposite demonstrated that the fillers caused a reduction in polymer matrix deformation due to an introduction of mechanical restraints. It has been reported that at the microstructural level, the volume of the ductile polymer phase may be confined by the surrounding stiff nanofillers phase, which may constrain the local deformation under stress, thereby impairing the elongation at break^53,54^. This restraining effect has been observed in this BaSO_4_/PLLA system. Hence, there appears to be a trade-off between the reinforcement effect and reduction in polymer’s ductility when rigid BaSO_4_ nanofillers were being introduced into PLLA.

#### Radiopacity

**Figure 3:**
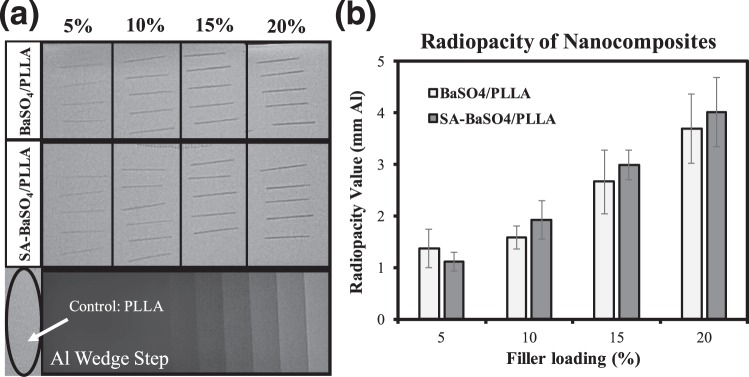
Radiopacity evaluation of the nanocomposites. (**a**) Digital radiographs of the nanocomposites with different filler loading, calibrated against the aluminum wedge step. (**b**) The radiopacity values of the samples expressed in mm Al. (n = 5 for each specimen). Error bar presents standard deviation.

The results for radiopacity evaluation of the nanocomposites are shown in Fig. 3. It can be seen that PLLA (control) is completely radiolucent and not visible under x-ray imaging. The addition of both non-functionalized and functionalized BaSO_4_ conferred radiopacity to the nanocomposite material (Fig. 3a) and the higher the filler loading, the higher the radiopacity value of the sample. From 5–15% filler loading, there were no significant difference between the radiopacity values of composites with BaSO_4_ or SA-BaSO_4_ (p > 0.05). This result demonstrated that the nanocomposite formulation can be to fabricate implants that are radiopaque, making visualization (using radiography) during procedure possible.

Current BRS (with the exception of REVA Medical’s FANTOM) have metallic (e.g. gold, platinum) radiopaque markers affixed onto the scaffold for visibility under x-ray. During scaffold deployment, the portion of a BRS with a marker may crack or stretch when stress is being applied, causing the markers to be dislodged in the process. A limitation of the current marker technology is that when viewed under fluoroscopy, the radiopaque markers do not provide good indication of scaffold expansion as they are usually only placed at the distal and proximal ends. During and after the procedure, the operator will not be able to assess scaffold expansion and lesion coverage accurately^1,55^. This also complicate retrieval in case of dislodgment of the scaffold from the delivery catheter. Hence, a material with adequate radiopacity can aid in addressing some of the imaging limitations of current polymeric BRS.

### Effect of functionalization on nanocomposite mechanical properties

**Figure 4:**
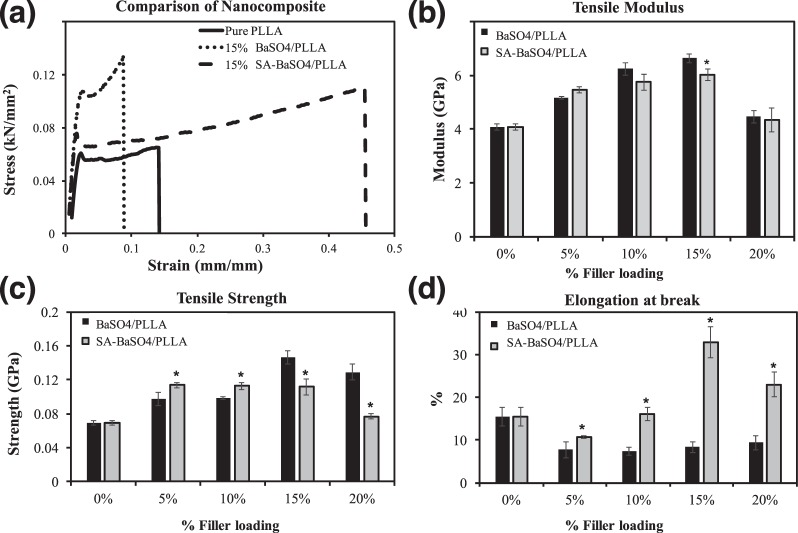
Tensile properties of non-functionalized BaSO_4_ and SA-BaSO_4_ filled PLLA. (**a**) Stress-strain curves of the pure PLLA and nanocomposite fibers. (**b–d**) Mean tensile modulus, strength and elongation at break of the nanocomposites. (n = 10) * indicates a statistically different value of the SA-BaSO_4_/PLLA compared to the BaSO_4_/PLLA at the same filler loading, based on one-way ANOVA (p < 0.05). Error bar presents standard deviation.

In this study, conjugation of stearic acid (SA) (less than 1 wt% of the functionalized BaSO_4_) to the surface of the nanoparticles had significant effect on the nanocomposite material as seen in the stress-strain curves (Fig. 4). In terms of modulus, functionalization did not affect the value significantly except at 15% loading (Fig. 4b). 15% filler loading gave the highest modulus for all the fillers-loaded PLLA, though the SA-BaSO_4_/PLLA had a lower modulus compared to non-functionalized ones. As for tensile strength, using the SA-BaSO_4_ resulted in higher strength at lower loading (<10%), after which the non-functionalized BaSO_4_/PLLA gave significantly higher tensile strength (Fig. 4c). One interesting observation is that the SA-BaSO_4_/PLLA nanocomposite material has significantly higher elongation at break across all the loading percentage, indicating an improvement in ductility compared to the non-functionalized system. SA-BaSO_4_/PLLA nanocomposite at filler loading of 15% onwards has a marked improvement in elongation at break compared to pristine PLLA material (Fig. 4d).

#### Tensile Modulus

The slight decrease of SA-BaSO_4_/PLLA modulus as compared to the non-functionalized BaSO_4_/PLLA can be ascribed to the effect of stearic acid on the filler/matrix interlayer, though the effect was only relatively significant at 15% filler loading. This observation is in agreement with literature, demonstrating that the interaction of fillers with matrix do not influence tensile modulus significantly. Since tensile modulus was determined at low stress, in the linear part of the stress-strain curve, filler de-bonding has not yet occurred and hence should not be significantly affected by functionalization^56–58^.

#### Tensile Strength

On the other hand, mechanical properties such as composite strength are more dependent on the interfacial interaction between fillers and matrix, thus warrants more discussion. The use of SA-BaSO_4_ led to better tensile strength at lower loading (<10%) compared to non-functionalized fillers. As the content of filler increases, the amount of lubricating stearic acid is increased accordingly in the interlayer, which may lead to lower interfacial stress transfer efficiency. The presence of stearic acid can lead to a plasticizer effect on the composite system, thereby decreasing the modulus compared to non-functionalized nanocomposites. Stearic acid has been observed to act as a plasticizer during the melt compounding process, which decrease the modulus slightly but improved the elongation at break^59,60^.

**Figure 5:**
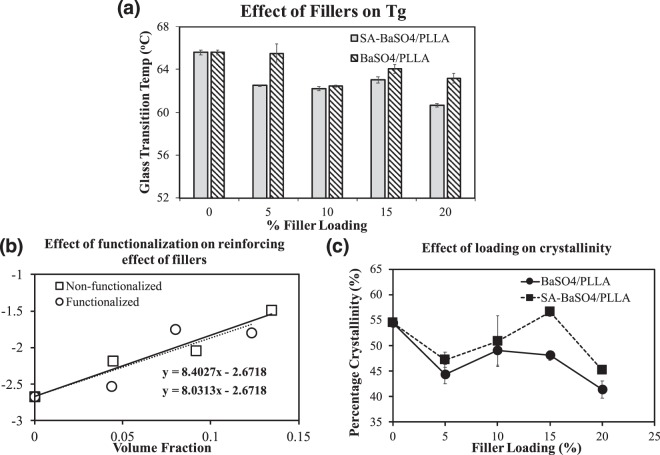
Effect of functionalization on nanocomposites based on DSC analysis and mathematical modeling. (**a**) Effect of filler loading on composite’s Tg in both non-functionalized BaSO_4_/PLLA and functionalized SA-BaSO_4_/PLLA. (**b**) Crystallinity of BaSO_4_/PLLA and functionalized SA-BaSO_4_/PLLA at different filler loading. * indicates a statistically different value of the SA-BaSO_4_/PLLA compared to the BaSO_4_/PLLA at the same filler loading, based on one-way ANOVA (p < 0.05). Error bar presents standard deviation. (**c**) Effect of SA functionalization on the reinforcing effect of the filler in BaSO_4_/PLLA composites, based on Equation 4. (The results for 20% filler loadings were omitted for both systems due to the decrease in tensile strength which were unable to be represented accurately by the mathematical models).

This plasticizer effect can be affirmed by the decrease in Tg and increased crystallinity of the functionalized SA-BaSO_4_/PLLA system (Fig. 5). The DSC analysis showed that with the addition of the functionalized SA-BaSO_4_ nanofillers, the decrease in Tg of the composite is significantly higher than that of the non-functionalized BaSO_4_/PLLA (Fig. 5a). The decrease of Tg of a polymer is attributed to the additional free volume caused by the plasticizer, which aids in facilitating segmental movement^61,62^. Figure 5b shows the crystallinity of the composites at different loading and the SA-BaSO_4_/PLLA have significantly higher crystallinity than non-functionalized BaSO_4_/PLLA from 15% fillers loading onwards. This result suggested that without functionalization, the fillers do not function as effective nucleating agent and have likely hindered the mobility of the polymer chains as discussed earlier. This observation has been reported in several polymeric composite systems, especially for fillers with poor interfacial adhesion to the matrix or a high filler loading^63–66^. It can be concluded that functionalized SA-BaSO_4_ had a plasticizer effect in PLLA.

To further examine the effect of filler loading on tensile strength of the composite, the strength of interfacial adhesion was estimated in this study using the following empirical equation by Pukanszky *et al*.^67^:

3 $${{\rm{\sigma }}}_{{\rm{c}}}={{\rm{\sigma }}}_{{\rm{m}}}.(1-{{\rm{V}}}_{{\rm{p}}}){(1+2{{\rm{.5V}}}_{{\rm{p}}})}^{-1}{\rm{.exp}}({{\rm{BV}}}_{{\rm{p}}})$$

where σ_c_ and σ_m_ refer to the tensile strength of the composite and matrix respectively, V_p_ is the volume fraction of the fillers and B is related to its relative loading bearing capacity, i.e. to the extent of reinforcement, which is dependent on interfacial adhesion. Rewriting Equation 3 into linear form yields Equation 4:

4 $$\mathrm{ln}\,[{\sigma }_{{\rm{c}}}.(1+2{{\rm{.5V}}}_{{\rm{p}}})\cdot {(1-{{\rm{V}}}_{{\rm{p}}})}^{-1}]={\mathrm{ln}{\rm{\sigma }}}_{{\rm{m}}}+{{\rm{BV}}}_{{\rm{p}}}$$

A graph of ln[σ_c_. (1 + 2.5V_p_). (1 − V_p_)^−1^] vs. V_p_ was plotted (Fig. 5c**)** and resulted in a linear correlation, the gradient (B) of the plot is proportional to the strength of interaction. Based on the Fig. 5c, it can be observed that the non-functionalized BaSO_4_ fillers (B = 8.40) had a slightly higher B value (dependent on interfacial adhesion) compared to SA-BaSO_4_ (B = 8.03). This could account for the lower mechanical properties of SA-BaSO_4_/PLLA compared to BaSO_4_/PLLA at the same filler loading. Hence, the decrease in composite strength at higher SA-BaSO_4_ loading could be due to the lubricating and plasticizing nature of stearic acid as mentioned earlier. The results agree with other mechanical studies on stearic acid functionalized nanofillers, where results have showed an improvement in filler dispersion due to lesser agglomeration but a reduced reinforcing effect of the fillers^31,38,39,68^. Experimental observations have demonstrated that the stearic acid modified filler did not improve the tensile modulus and strength of the polymers^69^.

#### Elongation at Break

**Figure 6:**
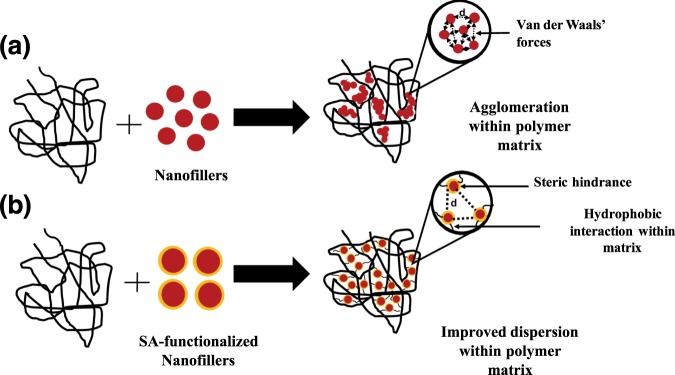
Schematic illustrating the effect of functionalization on filler dispersion in a polymer matrix. (**a**) Without functionalization, agglomeration of the nanofillers are evident due to high surface energies of the fillers. (**b**) With functionalization, targeted interactions can be established between the matrix and fillers, increasing the inter-particulate distance [d] and improving dispersion.

The significant improvement in elongation at break of the SA-BaSO_4_/PLLA could be explained in terms of interfacial viscoelastic deformation and matrix yielding (Fig. 4d)^70^. Stearic-acid functionalized nanofillers have been reported to achieve improved dispersion state due to lesser agglomeration. Without functionalization, the nanofillers aggregate due to their van der Waals’s bonding alignment and are expected to be in large clusters (Fig. 6a) within the polymer matrix. The interactions between the fillers cause a decrease in the inter-particulate distance [d] and result in clustering of the fillers. This leads to stronger inter-particulate adhesion, keeping the structural integrity of the large aggregates during deformation. The presence and size of these aggregates causes a reduction in matrix deformation by acting as mechanical restraints, thereby decreasing the composite’s ductility^53^. Rong *et al*. demonstrated that the decrease in ductility in the polymeric composite system suggested that matrix deformation is influenced by both interfacial interaction and dispersion state of the fillers. The decrease in ductility is found to be more pronounced at higher filler loading^20^.

**Figure 7:**
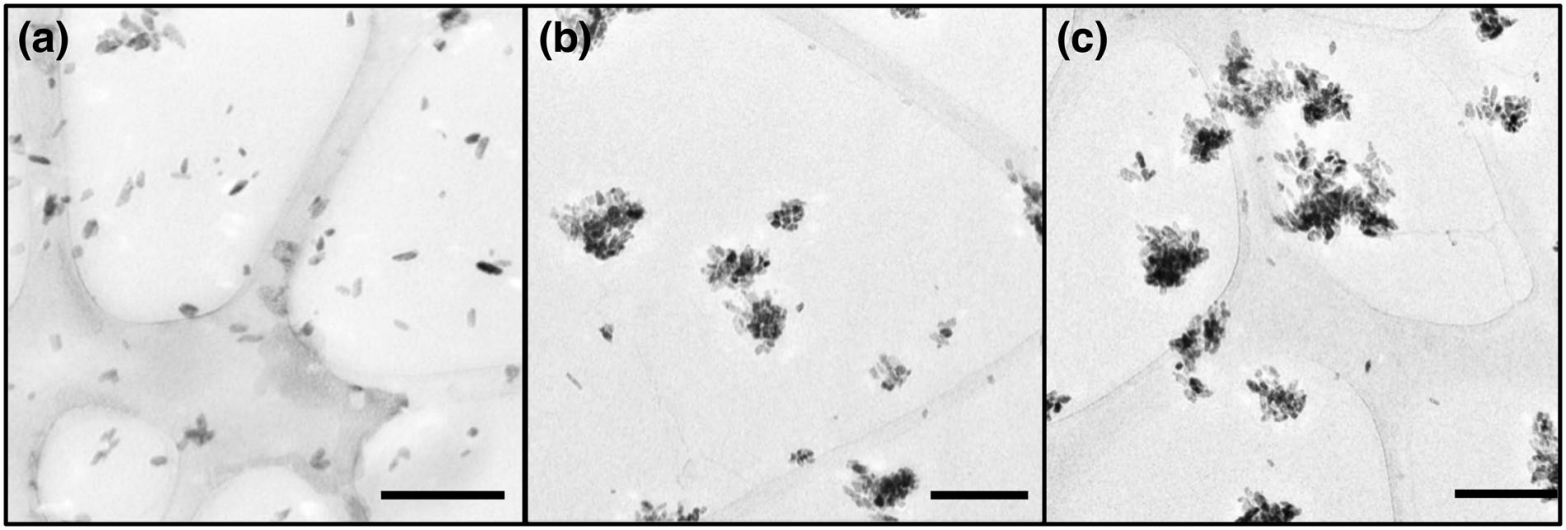
TEM images of the SA-BaSO_4_/PLLA nanocomposites with different filler loading: (**a**) 5%, (**b**) 15% and (**c**) 20%. The agglomeration of the nanofillers are less severe and it is possible to identify individual particle. Magnification: Scale bar: 200 nm.

On the other hand, stearic acid functionalization caused weakened filler/filler interactions, favoring a dispersion of smaller aggregates within the polymer matrix, increased the inter-particulate distance (d) between fillers as shown in the schematic in Fig. 6b^56,71^. Weakened interactions amongst the fillers and a smaller aggregate size leads to an increase in the number of fillers taking part in the deformation of the material. These explanations can be supported by the TEM images in Fig. 7 whereby the SA-BaSO_4_/PLLA had smaller clusters of fillers within the nanocomposites as compared to BaSO_4_/PLLA at the same loading percentage (Fig. 2). It can be observed that the SA-BaSO_4_ also exhibited aggregation at higher loading, reinforcing the detrimental effect of high filler loading on aggregation and mechanical properties. However, for the functionalized system, the resultant clusters are smaller in size and the fillers still maintained their distinct shape unlike the non-functionalized BaSO_4_, which appeared in larger agglomerated clusters.

Functionalization of nanofillers can be done to: (i) increase the hydrophobicity of the hydrophilic fillers in order to facilitate filler/matrix miscibility due to increased interaction between the two, (ii) prevent agglomeration of the fillers by introducing repulsive forces and (iii) improve the interfacial adhesion between the filler and matrix, thereby promoting a more effective transfer of stress, increasing the strength of the composite. Based on the results in this section, it can be concluded that the use of stearic acid to functionalize BaSO_4_ led to decreased agglomeration of the fillers but did not improve the interfacial adhesion between the filler and the polymer, which can be observed in the decrease in composite strength compared to the non-functionalized BaSO_4_. This could be due to the short chain length of stearic acid, which is considered too short to be effectively entangled within the polymer matrix^47,72^. Furthermore, it has been reported that the use of surfactant such as stearic acid has the plasticizer effect which weakens interactions between the fillers and polymer thus facilitating interface debonding^73^.

### Finite Element Analysis (FEA)

**Figure 8:**
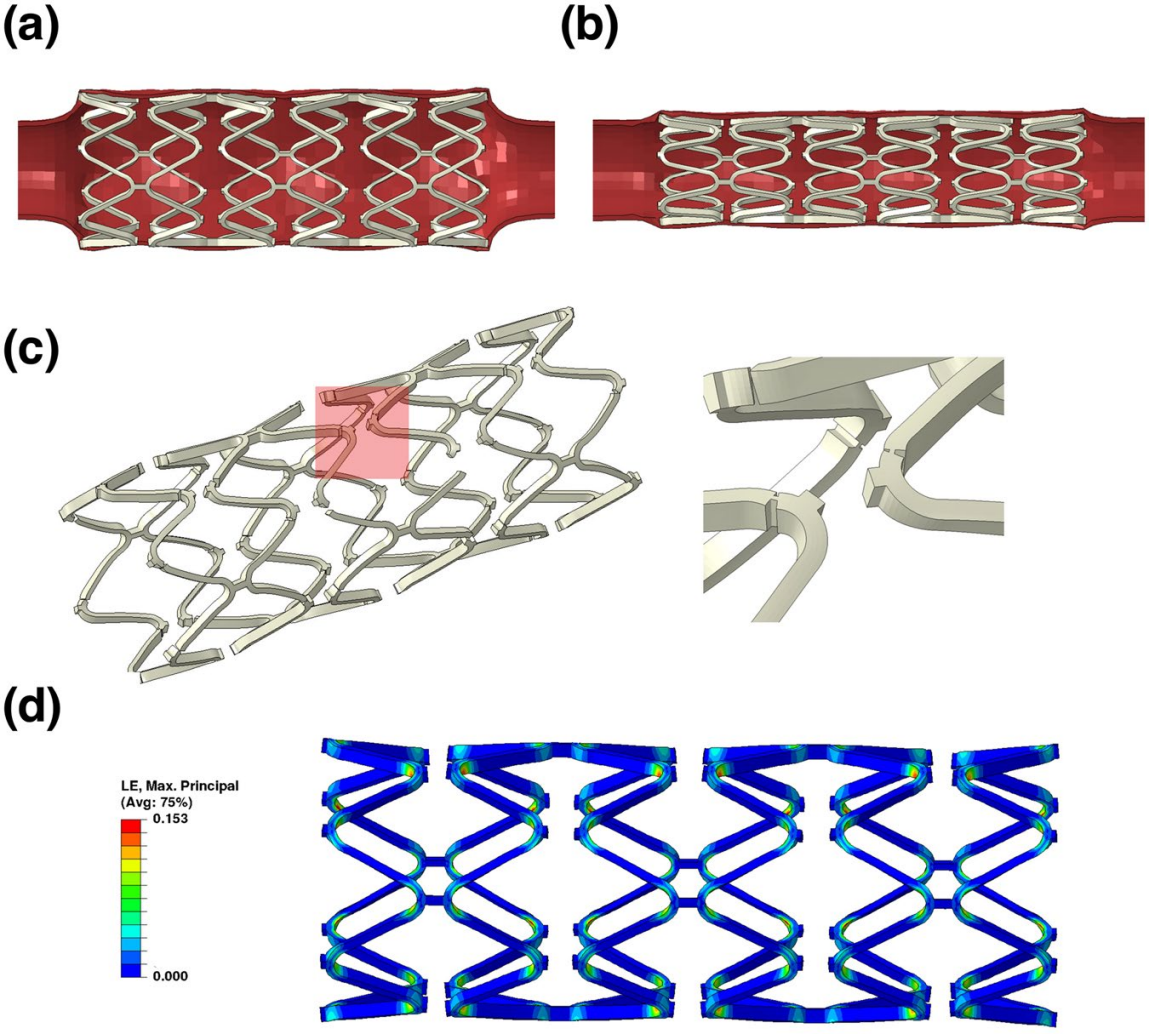
FEA of polymeric materials using a scaffold design with 150 μm strut thickness. (**a**) The expansion and (**b**) scaffolding of the 15% SA-BaSO_4_/PLLA BRS. (**c**) Scaffold fracture happened to 15% BaSO_4_/PLLA BRS during expansion and the marked part has been zoomed in to show the fractures. (**d**) The MP train contour of 15% SA-BaSO_4_/PLLA BRS at maximum expansion (PLLA-based BRS has a similar MP train contour, hence is represented by the same figure here).

Simulation work was done to compare the scaffolding ability of the scaffolds fabricated using different materials. The strut thickness with the SA-BaSO_4_/PLLA system was further modified with decreased thickness to evaluate the potential outcomes of using the nanocomposite material. The expansion and recoil of the 15% SA-BaSO_4_/PLLA scaffold with the tube is shown in Fig. 8a,b, and the PLLA scaffold displayed similar deformation. The 15% BaSO_4_/PLLA scaffold had several locations where the strain reached the elongation limit of the material during expansion, causing scaffold fractures as seen in Fig. 8c,d.

**Figure 9:**
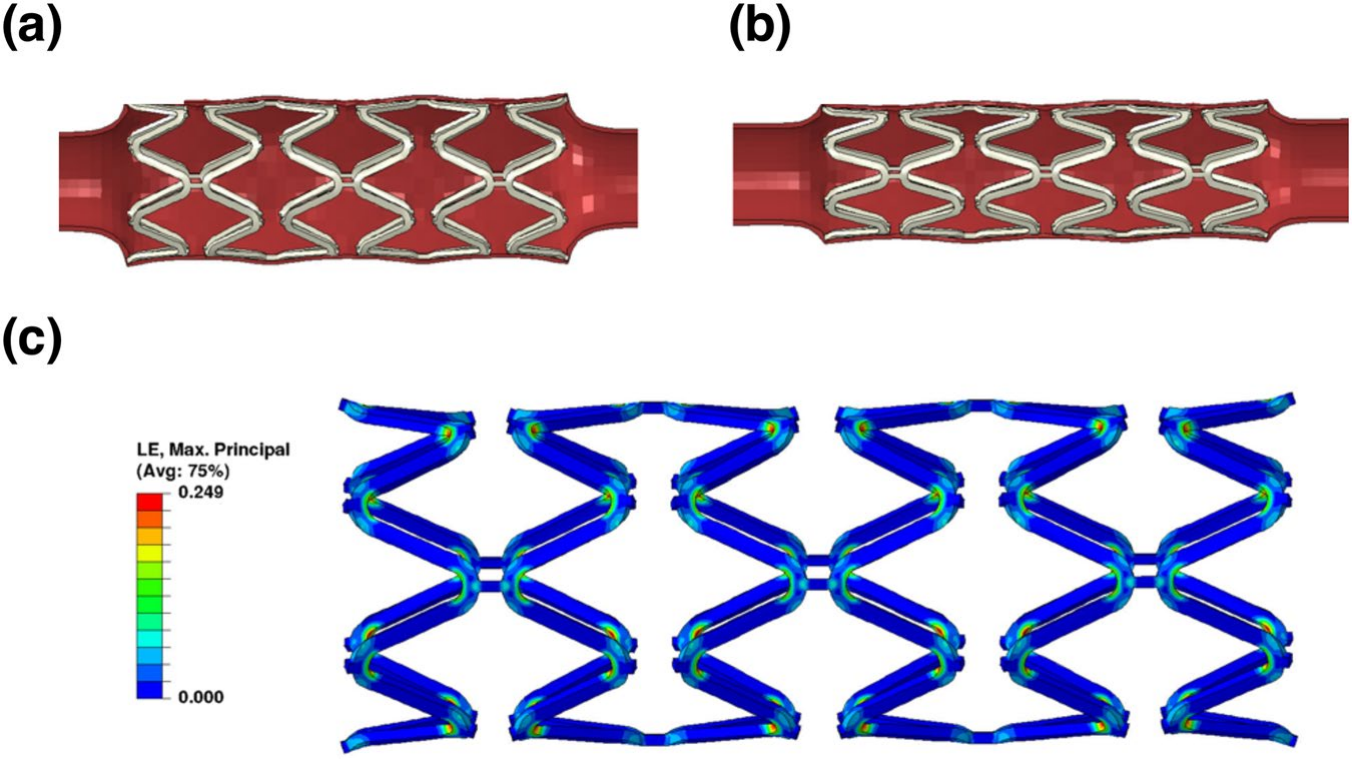
FEA of 15% SA-BaSO_4_/PLLA BRS with 100 μm strut thickness. (**a**) Expansion, (**b**) Scaffolding and (**c**) strain contour at maximum expansion of BRS with reduced strut thickness.

The averaged displacement of inner tube surface contacted with the scaffold is 0.16 mm for 15% SA-BaSO_4_/PLLA and 0.08 mm for PLLA stent respectively. The peak MP strain that the 15% SA-BaSO_4_/PLLA BRS experienced during expansion was 15.3% (Fig. 8d), which was far lesser than its elongation limit (Fig. 4d). 15% BaSO_4_/PLLA BRS had lesser peak MP stain (13.9%) compared to the functionalized BRS and a similar strain distribution, but its peak strain is near to its the elongation limit. The expansion and scaffolding of the 15% SA-BaSO_4_/PLLA BRS with reduced strut thickness (100 μm) is shown in Fig. 9a,b and the averaged tube displacement was 0.37 mm. During expansion, it had a peak MP stain of 24.9% (Fig. 9c), which is still below the elongation limit of the material.

To compare the influence of material properties on the BRS function, the FEA work evaluated three scenarios based on different stent materials and the results were aligned with the earlier mechanical testing outcomes. 15% BaSO_4_/PLLA BRS experienced fracture due to the low ductility of the material. On the other hand, the PLLA BRS barely survived the scaffold expansion with a scaffolding ability just half of the 15%SA-BaSO_4_/PLLA (considering the averaged displacement of inner tube surface contacting the scaffold).

Based on this simulation result, the 15% SA-BaSO_4_/PLLA BRS demonstrated reliable structure integrity during expansion and better scaffolding ability than PLLA and 15% BaSO_4_/PLLA. With enhanced material properties, the FEA data also showed that BRS fabricated from the functionalized nanocomposite material could potentially increase scaffolding by 130% (0.37 vs 0.16 mm) while reducing the thickness by 33% (0.1 vs 0.15 mm). Although the peak strain was increased by more than 60% (0.249 vs 0.153), the BRS still maintained its integrity and this could be attributed to the improved elongation limit of the SA-BaSO_4_/PLLA system.

Following the clinical lessons learnt from the BVS studies, there has been a concerted drive towards minimizing strut thickness. The newer generation of BRS has moved away from the original 150 μm design and has gone to lower than 100 μm. Thinner struts decrease protrusion and improve embedment of the struts, hence is expected theoretically to contribute to less flow disturbance compared to the thick struts of the current BVS, and possibly better endothelialization. Studies have shown that thinner strut BRS has better embedment of struts and lesser alteration of physiologic shear stress, thereby enhancing re-endothelialization^74,75^. The FEA work has provided important insights into the stress distribution and potential strut fractures of the BRS when deployed, and shown some of the potential mechanical advantages of the nanocomposite material in these aspects. This simulation can also aid in understanding the effect of strut thickness reduction on the expansion limit of the proposed nanocomposite materials.

## Conclusion

The mechanical properties of a nanocomposite BaSO_4_/PLLA material were modified using functionalization. The addition of rigid fillers such as nanosized BaSO_4_ had a reinforcement effect on the polymer as evidenced by the increase in tensile modulus and strength of the material, but ductility is decreased. The optimized formulation (15% BaSO_4_/PLLA) had an approximate 60% and 110% increase in tensile modulus and strength respectively, followed by 45% decrease in ductility. Functionalization of BaSO_4_ with stearic acid was shown to have decrease the agglomeration of the nanofillers, thereby improving the elongation at break of the composite significantly. Functionalization did not increase the modulus and strength of SA-BaSO_4_/PLLA as compared to BaSO_4_/PLLA (p > 0.05). Stearic acid as functionalizing agent was found to exert a plasticizer effect on PLLA, as evidenced by the DSC data and mechanical characterization. The BaSO_4_-filled composites were also radiopaque and could be visualized under x-ray. FEA demonstrated the potential benefit of using the nanocomposite material as a BRS candidate since the improved mechanical properties allowed for reduced strut thickness while maintaining structural support.

Future work will address some of the limitations of the current study in evaluating the material as a potential BRS candidate. For example, the nanocomposite material will be extruded into different thicknesses and laser-cut into a BRS prototype for further characterization such as radial testing and overexpansion evaluation. More FEA work will have to be conducted to evaluate other parameters such as different scaffold designs. The degradation rate and biocompatibility of the BRS will also need to be studied in order to understand its long-term behavior.

The use of polymeric composites reinforced with rigid fillers present an effective way to enhance the mechanical properties of the material for load-bearing devices. The fate and clearance of the fillers in the body after polymer degradation remains an area of scrutiny. BaSO_4_ is considered a poorly soluble low toxicity particle and its “particokinetics” is influenced by the particle size and route of exposure^76^. While several studies have been conducted on the clearance and toxicity of inhaled and intravenous (IV) injected BaSO_4_ nanoparticles, little data is available regarding the fate of implanted BaSO_4_^77–79^. It was found that for injected BaSO_4_ particles (~300 nm), fecal excretion was the dominant elimination pathway in a rat model. IV-injected BaSO_4_ was studied to understand the fate of circulating BaSO_4_ nanoparticles in the organs and it was demonstrated that a low concentration in the organs was achieved after 7 days in the animal model^77^. Previous work by Lämsä *et al*. had also explored the toxicity of a BaSO_4_/PLA stent in a rat model, reporting no adverse effect after 21 days^80^. More work has to be done in this area to better understanding on the clearance of the BaSO_4_ fillers after the degradation of the nanocomposite.

Presently, both the polymer-based and magnesium-based BRS platforms remain limited by their large profile and strut thickness, as compared to metallic DES. Research is being done to improve the properties of bioresorbable materials in order to reduce the strut thickness as seen from newer generation of BRS (e.g. Amaranth Medical’s FORTITUDE and APTITUDE). In conclusion, BaSO_4_/PLLA-based nanocomposites are good potential candidate materials for BRS with more desirable mechanical and radiopaque properties compared to PLLA alone.

## Materials and Methods

### Fabrication of nanocomposite polymeric material

PLLA (PURASORB PL inherent viscosity midpoint = 8 dl/g, Lot number: 0404002128) was purchased from Corbion (Netherlands) and employed as the base polymer. Non-functionalized BaSO_4_ nanoparticles is a product of Nanoshel (USA). The formulation of different nanocomposite samples fabricated in this study is shown in Table 1. The samples were fabricated using a twin-screw (Xplore Micro 5cc) microcompounder (Netherlands) and the polymer resin and nanoparticles were compounded at a temperature of 190 °C for 7 minutes before extrusion. The extrudates were cut into granules and fed back to the compounder for a second round of mixing for 5 minutes before being extruded. The nanocomposite fibers obtained from the extrusion were 180 ± 10 µm and were used for further testing and characterization.

**Table 1:** Formulations of different nanocomposite samples.

Sample	Nanoparticle	Filler Loading (%)
Control (PLLA)	No nanoparticles	—
BaSO_4_/PLLA	Non-functionalized BaSO_4_	5, 10, 15, 20
SA-BaSO_4_/PLLA	Functionalized SA-BaSO_4_	5, 10, 15, 20

### Transmission electron microscopy (TEM)

TEM (Libra 120 Plus, Carl Zeiss, Germany) was employed to examine the dispersion of the nanoparticles within the nanocomposite material. The extruded composite fibers were embedded in araldite epoxy resin (Ted Pella, USA), cured at 60 °C for 24 hours and sectioned into 100 nm slices using ultramicrotomy. The slices were floated on a Formvar-coated copper TEM grid from deionized water and visualized using the TEM. The volume fraction of the nanocomposite material was determined from the automatic threshold method using Image J software. In this method, color-based thresholding was used to segment the fillers from the polymer matrix. The “Analyze” tool from the software will calculate the integrated densities (Area (No. of pixels) x Total Intensity of the pixels) of the different regions. This provides an estimation for the volume fraction of the fillers in the nanocomposite material. The volume fraction of the fillers is presented as an average of 10 TEM images per sample and 3 samples were used for each formulation. Image analysis was also performed using the same software to determine the size distribution of the agglomerated BaSO_4_ nanoparticles within the PLLA matrix. A total of 120 nanoparticles were measured.

### Differential scanning calorimetry (DSC)

A Perkin- Elmer DSC 8000 was used to study the thermal properties at a heating rate of 10 °C/min. The samples were cut into smaller pieces to improve contact with the sample pan. The Tg was determined as the point of half heat capacity extrapolated and the melting temperature (Tm) was determined as the peak temperature. The crystallinity of the material was calculated from the fusion enthalpy of melting (ΔH_m_), which was obtained from the DSC melting curve. Based on literature, the theoretical heat fusion of 100% crystalline PLLA was employed to be 93.1 J/g. The crystallinity of the PLLA in this work was calculated by^81^:

5 $$ \% \,{\rm{Crystallinity}}=({{\rm{\Delta }}{\rm{H}}}_{{\rm{m}}}/93.1\times {{\rm{W}}}_{{\rm{p}}})\times 100 \% $$

where ΔH_m_ is the enthalpy of fusion for the sample in J/g. an W_p_ is the weight fraction of PLLA in the composites. Triplicates were done for each sample to ascertain the reproducibility of the results.

### Mechanical testing of nanocomposite polymeric material

All nanocomposite materials were subjected to tensile testing using an MTS C42 Test System (MTS Systems, MN, USA). A 50 N load cell was used for the testing and all samples were pulled at a constant crosshead speed until failure. The extension rate was set at 0.1 mm/min and the stress-strain curves were obtained using the MTS software. The tensile modulus, the ultimate tensile strength (UTS) and elongation at break were obtained from the stress-strain curves. The values were averaged from 10 samples for each sample. The test specimens (nanocomposite fibers) were all standardized to be 180 ± 10 µm in diameter and 40 mm in length (for the testing length) after being mounted onto the MTS.

### Radiopacity measurement

For radiopacity evaluation, five samples per concentration (extruded fibers with 1.00 ± 0.05 mm in diameter and 20 ± 2 mm in length) were used. The samples were placed adjacent to a calibrated aluminium step wedge (Biomedia, Singapore) with 3.2-mm increments and imaged according to the adapted protocol^82^. A standard X-ray machine (Philips Clarity FD20) was used to irradiate X-rays onto the specimens using an exposure time of 4 ms at 76 mA and a cathode-target film distance of 100 cm. The tube voltage was set at 50 ± 5 kV. The radiographs were processed (AGFA Enterprise Imaging System), and a digital image of the radiograph was obtained. The grey pixel value on the radiograph, of each step in the step wedge was determined using an imaging programme, ImageJ (NIH, USA). Numbers between 0 and 255 with 0 representing pure black and 255 pure white were assigned accordingly. The equation of the trend line in the graph of aluminium thickness vs. grey pixel value can then be used to calculate the pixel value of a sample based on the sample thickness in mm of aluminium. The grey pixel values of the samples were computed ImageJ and the obtained equation to calculate the equivalent radiopacity expressed in mm of aluminium.

### Statistical analysis

All mechanical tests were done in 10 repetitions for each type of nanocomposite samples and numerical data were analyzed using standard analysis of variance (ANOVA) technique and statistical significance was considered at p < 0.05.

### Finite Element Analysis

**Figure 10:**
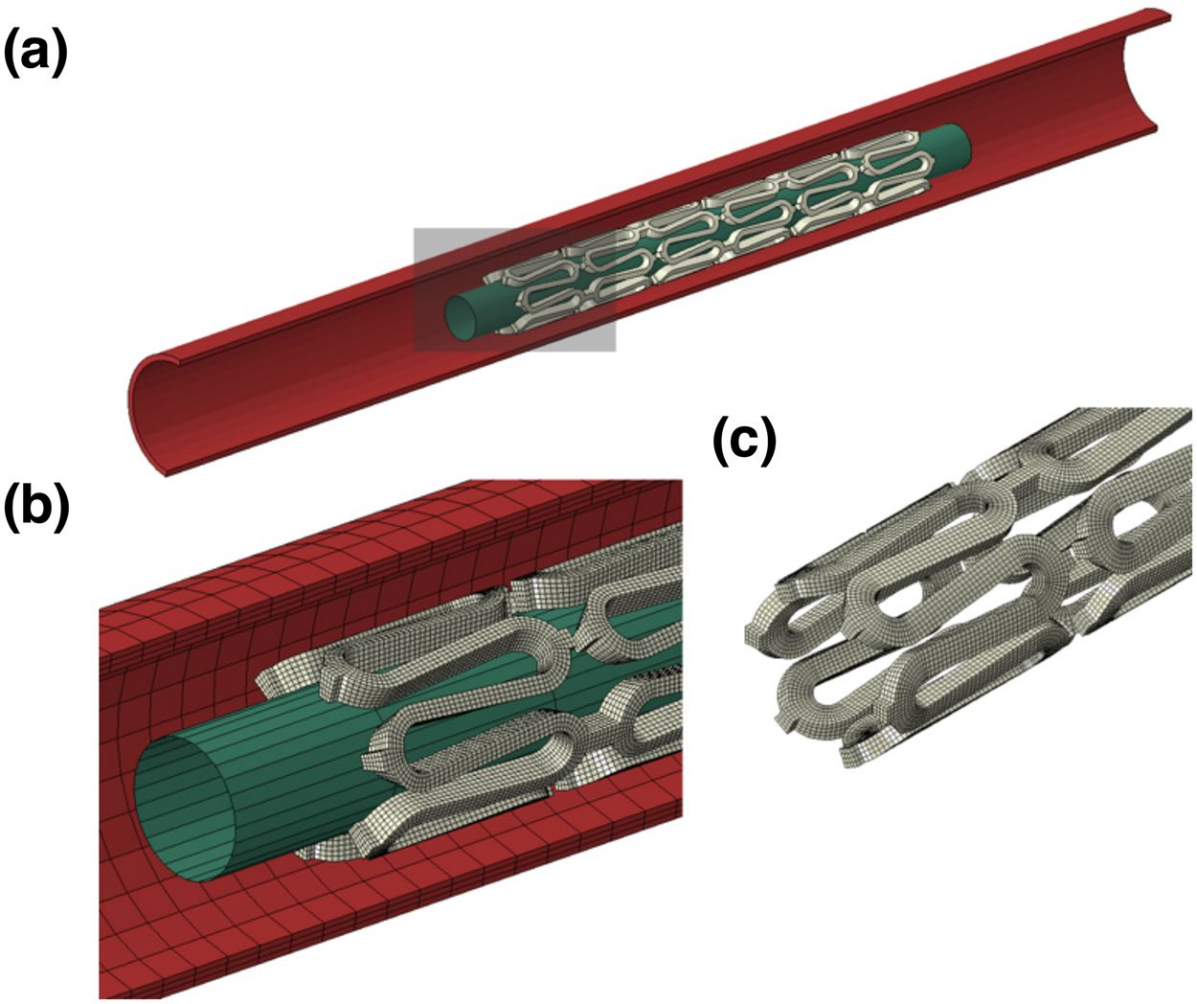
The FEA model employed in this study consists of a stent, a rigid balloon, and a silicon rubber tube (**a**) and the marked part was amplified to show the (**b**) meshing of the model and the (**c**) shape and meshing of the scaffold with reduced strut thickness.

In this study, the simulation involved the implantation of a scaffold into a silicon rubber tube to examine if the device can maintain its integrity during expansion. The whole model employed in this simulation is shown in Fig. 10a,b. The generic scaffold design has a length of 8 mm, an outer diameter of 1 mm, a thickness of 0.15 mm (150 μm) and strut width of 0.9 mm with six rings connected by two links in-between. The material properties of the three materials (PLLA, 15% BaSO_4_/PLLA and 15% SA-BaSO_4_/PLLA) were assigned to the scaffold model for three simulation scenarios.

The silicon rubber tube has a thickness of 0.1 mm, an inner diameter of 1.8 mm and a length of 20 cm (E = 1.5 MPa and v = 0.49). The rigid balloon has a diameter of 0.7 mm and a length of 10 mm. The modified scaffold design (Fig. 10c) has a thickness of 0.1 mm (100 μm) and width of 0.15 mm (the corresponding balloon has an increased diameter of 0.8 mm). The scaffold and tube model were meshed with hexahedral solid elements with rigid balloon surface elements. A sensitive analysis has been applied to ensure the elements density is enough. Two interactions, between the inner scaffold surface and rigid balloon, and between outer scaffold surface and inner tube surface, were established to simulate the contacts between scaffold and rigid balloon, and between scaffold and tube.

Quasi-static simulation was carried on using the finite element code ABAQUS/Explicit (Dassault Systèmes Simulia Corp., Providence, RI). In each of the four scenarios, the rigid balloon was expanded until the outer stent diameter reached 3 mm, followed by the recoiling of the balloon to its original shape, leaving the BRS supporting the tube by itself. For boundary conditions, the tube was fixed in all directions at its two ends and the rigid balloon can only expand and recoil in radial direction.

A material fracture mechanism was considered in the simulation to evaluate if the materials could endure the severe deformation during scaffold expansion. When the strain of a scaffold element reaches the elongation limit of the material assigned, the element will be considered as having failed, and therefore deleted from the model. For the scenarios without scaffold element failure, the averaged displacement of the inner tube surface contacted with the scaffold was calculated to evaluate the scaffolding ability of the scaffold; meanwhile, the maximum principal (MP) strain of the scaffold during expansion was also evaluated. As required by the FEA code, the strains applied in the simulation and shown in FEA results were true strains (logarithmic strains, LE).

## Data Availability

The data that support the findings of this study are available within the paper.
